# Usefulness of the Duke Activity Status Index to Assess Exercise Capacity and Predict Risk Stratification in Patients with Pulmonary Arterial Hypertension

**DOI:** 10.3390/jcm12082761

**Published:** 2023-04-07

**Authors:** Haofeng Zhou, Yu Wang, Weiya Li, Lifang Yang, Yingxue Liao, Mingyu Xu, Caojin Zhang, Huan Ma

**Affiliations:** 1 Guangdong Cardiovascular Institute, Guangdong Provincial People’s Hospital, Guangdong Academy of Medical Sciences, Guangzhou 510000, China; 2 School of Medicine, South China University of Technology, Guangzhou 510000, China; 3 Guangdong Provincial People’s Hospital, Guangdong Academy of Medical Sciences, Southern Medical University, Guangzhou 510000, China

**Keywords:** pulmonary arterial hypertension, exercise capacity, Duke Activity Status Index, cardiopulmonary exercise testing

## Abstract

Exercise capacity is an important component of risk assessment for pulmonary arterial hypertension (PAH). We investigated the association of the Duke Activity Status Index (DASI) with peak oxygen consumption (peakVO_2_) and explored whether the DASI can discriminate the high-risk individuals in patients with PAH, according to peakVO_2_ < 11 mL/min/kg. A total of 89 patients were evaluated using cardiopulmonary exercise testing (CPET) and DASI. The correlation between the DASI and peakVO_2_ was measured by univariate analysis, and a receiver operating characteristic (ROC) curve analysis was conducted. The DASI was correlated with peakVO_2_ in the univariate analysis. The ROC curve analysis revealed that the DASI had a discriminative value for identifying the individuals with a high risk in PAH patients (*p* < 0.001), with an area under ROC curve (AUC) of 0.79 (95% CI: 0.67–0.92). Similar results were observed in patients with PAH associated with congenital heart disease (CHD–PAH), (*p* = 0.001), with an AUC of 0.80 (95% CI: 0.658–0.947). Therefore, DASI reflects exercise capacity in patients with PAH and has good ability to discriminate patients with a low risk and a high risk, and it may be included in the risk assessment of PAH.

## 1. Introduction

Pulmonary arterial hypertension (PAH) is an increase in pulmonary circulation pressure caused by structural or functional changes in the pulmonary artery, affecting about 1% of the global population [1,2]. Idiopathic PAH (IPAH) is the most common subtype in economically developed countries, whereas PAH associated with congenital heart disease (CHD–PAH) represents an important subcategory in developing countries [3,4]. Due to the remodeling of the pulmonary vascular bed and an elevation of pulmonary arterial pressure, leading to progressive right heart dysfunction and exercise intolerance [5,6], impaired exercise capacity is a frequent and cardinal in individuals with PAH, which not only has an impact on patients’ quality of life, but also correlates with a worse prognosis [7,8]. Therefore, exercise capacity is now recognized as an issue of major clinical importance in the assessment and management of PAH.

The cardiopulmonary exercise test (CPET) and the 6 min walking test (6MWT) are two main measures of exercise capacity in PH centers. CPET is considered the current gold standard modality to assess the exercise capacity in humans [9]. Peak oxygen consumption (peakVO_2_), one of the predominant parameters derived from CPET, provides objective measures related to exercise tolerance. Each 3.5 mL/kg/min increase in peakVO_2_ is associated with a 16% improvement of the survival rate across populations [10]. Robust prognostic evidence for peakVO_2_ of PAH patients has been found in three studies [11,12,13]. PeakVO_2_ < 11 mL/min/kg is used to determine high-risk status, representing an estimated 1-year mortality more than 20%, in the 2022 ESC/ERS guidelines for pulmonary hypertension [2]. The 6 min walk test (6MWT) represents a reliable and simple method of evaluating exercise capacity and is routinely used to assess PAH patients. The 6 min walking distance (6MWD) > 400 m is an indicator for a better prognosis of PAH, and a deterioration in 6MWD has a great predictive value on key clinical outcomes [14,15].

However, both tests have some limitations. The use of CPET requires specialized expensive equipment and skilled personnel, with limited availability in most facilities. Although major equipment is not required for conducting the 6MWT, a 30 m long interrupted corridor and a trained medical team are necessary. Furthermore, the duration of both tests usually exceeds 30 min due to the pretest preparation and post-test resting. Therefore, the requirements of equipment, staff, and time make both tests inconvenientto deliver in those busy clinical settings with limited medical resources, particularly in developing countries. In such situations, a more practical tool to assess the exercise capacity of individuals with PH may be required. The Duke Activity Status Index (DASI) is a brief and self-administered questionnaire, reflecting on exercise capacity based on patients’ yes or no answers to 12 questions related to their daily activities [16]. The DASI has been shown to significantly correlate with peak VO_2_ in patients with cardiovascular disease, cancer patients, and patients undergoing general surgical, and it provides valuable information on disease severity, effects of rehabilitation, and prognosis in cardiovascular patients [17,18,19,20,21]. The DASI score of 26.2 was demonstrated to have the ability to predict a 5-year mortality of heart failure patients using the receiver operating characteristic (ROC) curve, with an area under the ROC curve (AUC) of 0.69 [22]. The DASI score of 34 is also used to identify preoperative patients with a high cardiac risk, recommended by the ACC/AHA and ESC/ERS guidelines for patients undergoing noncardiac surgery [23,24,25].

A previous study has demonstrated a correlation between the DASI and 6MWD in patients with pulmonary hypertension, preliminarily illustrating the validity of the DASI in measuring exercise capacity [26]. However, there is little research on the relationship between the DASI and peakVO_2_ in PAH patients. Against this background, we hypothesized the following: (1) the DASI would significantly correlate with exercise capacity measured by peakVO_2_ and 6MDW in the PAH population; (2) the DASI would reliably identify high-risk and non-high-risk patients according to peakVO_2_ < 11 mL/min/kg and peakVO_2_ > 11 mL/min/kg, according to the ESC/ERS guidelines, so as to provide a cost-free and simple surrogate method for clinicians and patients to assess exercise capacity, especially in resource-limited settings.

## 2. Methods

### 2.1. Study Setting

This single-center observational cross-sectional study was conducted at the outpatient department of Guangdong Provincial People’s Hospital, a tertiary-care cardiovascular health center in south China. The study protocol was approved by the ethics committee of Guangdong Provincial People’s Hospital. All procedures were conducted according to the Declaration of Helsinki, and informed consent was obtained from participants or their guarantees prior to any initiation of study procedures [27].

### 2.2. Participants and Recruitment

The inclusion criteria of the study were (1) diagnosis with PAH by right-sided heart catheterization measurement of arterial pulmonary pressures > 25 mmHg; (2) aged 18 years or older; (3) able to complete CPET and 6MWT. Patients were excluded if they had one of the following conditions: (1) severe comorbidity (e.g., untreated left heart disease and uncontrolled hypertension); (2) unable to communicate; and (3) unwilling to sign the informed consent. The study recruited outpatients at the cardiovascular clinic from August 2021 to October 2021. The potential participants were offered a brief introduction to the study, and they had the opportunity to ask questions directly. Those willing and capable to participate subsequently signed the written informed consent after confirming their study eligibility.

### 2.3. Procedure

**Figure 1:**
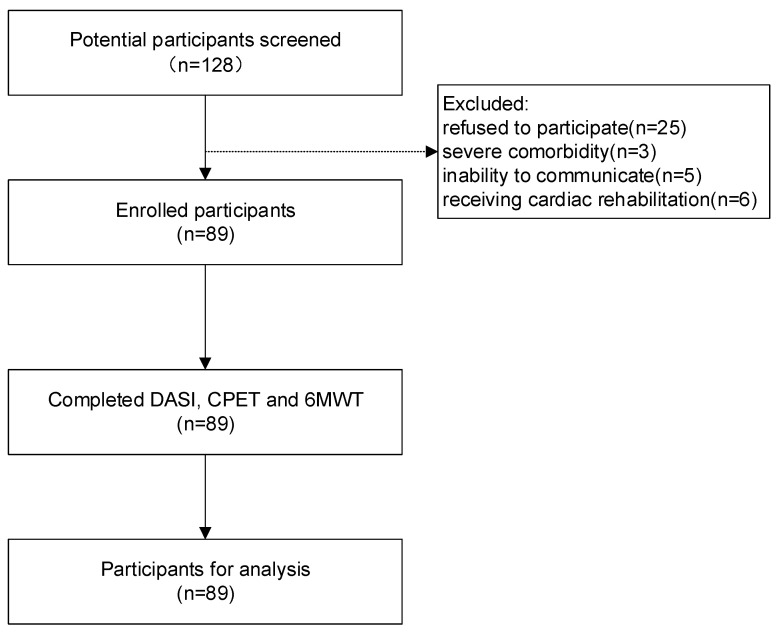
Flow diagram of the study.

The study flow diagram is shown in Figure 1. After enrollment, patient demographics, such as height, weight, educational level, history of smoking, and WHO functional class (WHO-FC), were gathered through face-to-face interviews. Then, relevant clinical information was collected by interviews and reviewing the medical records, including PAH subsets, recent echocardiography, and hemodynamic parameters. Participants were scheduled to accept evaluation of exercise capacity using the DASI, CPET, and 6MWT. Three tests of each participant were finished on the same day in outpatient clinic.

### 2.4. Assessment of Exercise Capacity

Duke Activity Status Index: The DASI is a self-administered questionnaire that captures physical functional status, consisting of 12 items about the patient’s ability to perform common daily living activities about personal care, ambulation, housework, yard work, and sexual function, as well as recreational activities [16]. Participants may select yes or no as the answer to each item, which was weighted based on the metabolic cost associated with the activity. The total score, ranging from 0–58.2, was obtained by adding the weighted scores for the 12 items, with higher scores indicating better activity status. The DASI has previously been used to demonstrate functional exercise capacity of Chinese patients [28]. In the current study, the DASI was adapted to the Chinese language and culture, and participants were instructed to finish the questionnaire through interviews.

Cardiopulmonary Exercise Test: CPET was performed on a bicycle ergometer using a ramp protocol with 12-lead ECG monitoring, and respiratory gas exchange was analyzed breath-by-breath by a metabolic cart. Patients began pedaling at a load of 20 W, and the resistance was increased by 10–15 W every minute and maintained a pedaling speed of 60 ± 5 revolutions per minute. During the test, heart rate, blood pressure, and oxygen saturation were monitored continuously. The test stopped after patients achieved the targeted workload; either they failed to pedal at the targeted speed or experienced dyspnea, chest pain, dizziness, and hypotension. PeakVO_2_ was derived from respiratory gas analysis during maximal exercise testing and recorded in per kg body mass per min (mL/kg/min). This regimen has been utilized to measure peakVO_2_ among myocardial infarction patients in our prior study [29].

The 6-Minute Walk Test: The 6MWT was conducted in a corridor of 30 m in length on flat, hard ground in accordance with ATS guidelines [30]. Patients were instructed to walk back and forth as fast as they could in 6 min. Encouragement was provided once every minute. Oxygen saturation, heart rate, and symptoms were continuously monitored to keep participants safe, and adequate encouragement was provided once per minute. Rating of perceived exertion was assessed by Borg CR-10 scale after completing the test to determine subject’s effort [31]. The 6MWD was measured in meters.

### 2.5. Data Analysis

The sample size calculation was based on association between the DASI score and peakVO_2_ in patients with PAH. As there was research on the association between the two in this cohort, we hypothesized that we would detect a relationship between the DASI score and peakVO_2_ with a correlation coefficient of at least 0.3 in patients with PAH, considering a correlation coefficient of 0.38 was reported in heart failure patients. Thus, we aimed to recruit 82 participants to provide a power of 80% for detecting such relationship at the two-sided alpha level of 0.05.

Data analysis was performed using the IBM SPSS software version 20 (IBM Corp., Armonk, NY, USA). There were no missing values in the main variables, and therefore, no imputation was performed. Continuous variables are presented as mean ± standard deviation, and categorical variables are shown as numbers and percent (%). Comparisons between groups were using the Mann–Whitney U test, the Student’s t-test, or the Pearson’s chi-square test, as appropriate. To assess the relationship between the DASI score and exercise capacity, a univariate linear regression model was performed using the DASI score as the independent variable, with peakVO_2_ and 6MWD as the dependent variables. ROC curve analysis with calculation of the AUC was constructed to evaluate the ability of the DASI to discriminate patients with PAH in different risk statuses. The AUC values were classified as follows: AUC < 0.5 suggests poor predictive ability, 0.7 ≤ AUC < 0.8 indicates good predictive ability, and 0.8 ≤ AUC < 0.9 suggests excellent predictive ability. Youden’s index was used to determine the best cut-off value that met with the criterion of maximum sensitivity and specificity. For subgroup analysis, patients were classified into high-risk or non-high-risk groups according to the cut-off value for the DASI; then, two groups were compared in terms of WHO-FC using chi-square test. For all tests, a *p*-value < 0.05 was considered statistically significant.

## 3. Results

A total of 128 potentially eligible participants were screened. Twenty-five patients refused to participate. Three patients with severe comorbidities were excluded, and five were not eligible due to an inability to communicate. To avoid bias, six patients already receiving a cardiac rehabilitation program including aerobic exercise were excluded. Thus, a total of 89 PAH patients were enrolled in the study. Patient demographics and clinical characteristics are described in Table 1. In 89 patients, 20 were diagnosed with IPAH, 62 were PAH associated with congenital heart disease, and 7 were PAH associated with other causes. Most patients were female (83.1%), had a mean age of 34.53 years, and the BMI was 20.29 kg/m^2^. The mean duration of all patients was 5.73 years since the diagnosis was confirmed. Most patients presented with a WHO-FC I/II; the remaining six individuals demonstrated WHO-FC III. The average of the DASI score was 36.47. The mean peakVO_2_ and 6MWD was 15.22 mL/min/kg and 428.1 m, respectively. There were 20 patients whose peakVO_2_ < 11 mL/kg/min, indicating a high-risk status according to the 2022 ESC/ERS guidelines for pulmonary hypertension. Except for BMI, patients with different PAH subsets did not show any significant differences in the demographic and clinical characteristics, as well as exercise capacity.

**Figure 2:**
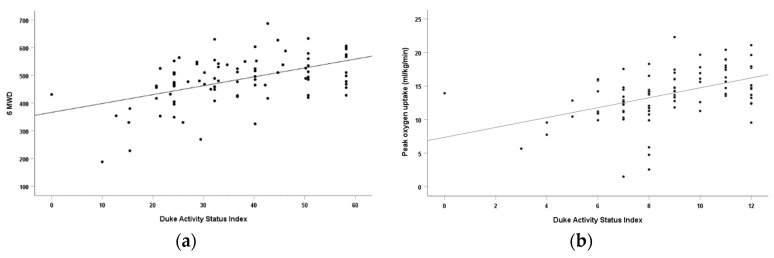
(**a**) The association between the DASI score and peakVO_2_ in PAH patients. (**b**) The association between the DASI score and 6MWD in PAH patients.

A univariate linear regression analysis was conducted to determine the relationship between the DASI score and peakVO_2_ and 6MWD. In PAH patients, the DASI score was an independent predictor for both peakVO_2_ and 6MWD, explaining 22% of the variance in peakVO_2_ and 25% of the variance in 6MWD (*p* < 0.001). Additionally, the DASI score significantly correlated with the peakVO_2_ (r = 0.467) and 6MWD (r = 0.501), indicating moderate relationships between the DASI and exercise capacity (Table 2). Scatterplots for the association between the DASI score and peak VO_2_ and 6MWD are presented in Figure 2. Similar results were also observed in patients diagnosed with CHD–PAH.

**Figure 3:**
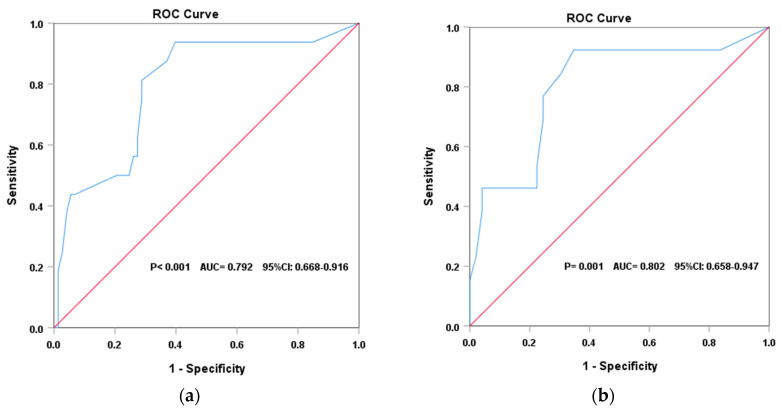
(**a**) Discriminative ability of the DASI to identify high-risk patients with PAH. (**b**) Discriminative ability of the DASI to identify high-risk patients with CHD–PAH.

The ROC curve analysis revealed that the DASI had a good predictive value for identifying patients of different risk status. In PAH patients, the DASI score showed a good discriminative value for identifying high-risk patients from those who were non-high-risk (*p* < 0.001), with an AUC = 0.792 (95% CI: 0.668–0.916). The optimal cut-off value was 33.8, which had a sensitivity of 0.91 and a specificity of 0.65. In the CHD–PAH population, the DASI score had an excellent predictive ability in discriminating patients in a high-risk status from others (*p* = 0.001). The AUC was 0.802 (95% CI: 0.658–0.947), and the best cut-off value was 33.8, with a sensitivity of 0.92 and a specificity of 0.0.64 (Figure 3).

For the subgroup analysis, all patients were classified into high-risk and non-high-risk groups according to the cut-off value of 33.8 for the DASI; then, the distribution of WHO-FC was compared between the groups. Patients with a DASI score > 33.8 presented better WHO-FC than patients whose DASI score < 33.8 (*p* = 0.026), supporting the score of 33.8 as a cut-off value for risk stratification (Table 3). Furthermore, the cut-off value of 33.8 classified patients in WHO-FC II as different risk status, with 32 high-risk patients and 29 non-high-risk patients.

## 4. Discussion

This study aimed at investigating the relationship between the DASI and exercise capacity and establishing the DASI cut-off value to discriminate high-risk PAH patients according to peakVO_2_ < 11 mL/min/kg by CPET. A total of 89 PAH patients were enrolled, 20 of whom were classified as high-risk according to peakVO_2_. The proportion of high-risk patients was close to the previous study [12]. Our results show that the DASI score was an independent predictor for both peakVO_2_ and 6MWD in PAH patients. The ROC analysis demonstrated that the DASI had a relatively good ability for risk stratification. The DASI score of 33.8 was sensitive and specific to discriminate high-risk patients from those who were non-high-risk. The DASI was originally developed for the evaluation of patients with cardiovascular diseases, and, therefore, a significant correlation with peakVO_2_ is well demonstrated in multiple cardiovascular cohorts, with the correlation coefficients ranging from 0.31 to 0.71 [19,32,33,34,35,36]. Furthermore, the DASI score is associated with exercise capacity in patients with chronic obstructive pulmonary disease, patients with cancer, and patients undergoing general surgery [17,21,37]. The strong relationship between the DASI and 6MWD in PH patients has been reported by others [26]. In our study, the DASI is significantly associated with peakVO_2_ by CPET and 6MWD, confirming that the DASI is a reliable and valid method to reflect exercise capacity of PAH patients.

In the literature, the DASI provides independent prognostic value for mortality in multiple cardiovascular diseases. Several studies demonstrated that a lower DASI score conferred a worse prognosis in patients with ischemic heart disease [32,38,39]. For example, the results of the WISE study suggest that 67% of all-cause deaths or myocardial infarctions occurred in subjects with a DASI < 16.5 [39]. In stable heart failure patients, the lowest DASI score quartile predicted a 2.6-fold increase in the 5-year mortality risk when compared to the highest DASI score quartile, after adjusting for BNP, eGFR, and LVEF [22]. Additionally, the DASI predicts the short- or medium-term progression from stage-B to overt heart failure [40]. Hence, the DASI may be an independent prognostic predictor of PAH. In the 2022 ESC-ERS guidelines for pulmonary hypertension, patients stratified into the high-risk group represent estimated 1-year mortality rates > 20, and peakVO_2_ is one of the modifiable variables to help identify patients having a high risk of death. Taking these into consideration, we classified patients at a high or non-high risk according to peakVO2 and subsequently analyzed the ability of the DASI to discriminate between the two groups. This study found the DASI has an excellent ability to discriminate PAH patients into high-risk or non-high-risk groups, and a DASI score of 33.8 was the best cut-off value for classifying patients at different risks, with satisfactory sensitivity and specificity. In another study, a DASI score of 26 was used to discriminate PAH patients with a better or worse long-term prognosis according to 6MWD of 400 m, having 74% sensitivity and 88% specificity [23]. The difference of the cut-off value may be explained by different degree impairments of exercise capacity in the PAH subjects enrolled. The mean 6MWD and DASI scores were 427.1 m and 27.99, respectively, in the previous study, indicating that those patients have a worse exercise capacity compared with the patients in the current study. In addition, patients were discriminated into a better and worse prognosis according to a 6MWD of 400 m, which led to a lower cut-off value in the DASI naturally. Nevertheless, the important point is that the DASI is able to provide a cut-off value for risk stratification in patients with PAH.

The WHO-FC is another key part of evaluating patients with PAH, providing valuable information for determining disease severity, and patients of WHO-FC I/II are expected to have a better prognosis [2]. We conducted a subgroup analysis using the WHO-FC to support the utility of the DASI. Patients were classified into high-risk and non-high-risk groups according to the DASI of 33.8. The proportion of WHO-FC was significantly different in these two groups, and the cut-off value helped to discriminate patients of WHO-FC II into different risk status, supporting the utility of the DASI for risk stratification.

The ESC/ERS guidelines for pulmonary hypertension highlight the risk stratification of patients as an increasing priority at both baseline and follow-up. Patients are classified into low-, intermediate-, and high-risk groups based on a comprehensive assessment including of symptoms, exercise capacity, blood biomarkers, imaging, and hemodynamic measures. The exercise capacity provides valuable information for determining disease severity and quality of life. CPET and 6MWT have requirements for equipment and medical personnel [26,37], and both may also be costly for most patients in developing countries, since many PAH medications are expensive with a low reimbursement proportion from medical insurance. Our study suggests that the DASI is significantly correlated with exercise capacity and has a good predictive ability of risk stratification in PAH patients, also in patients with CHD–PAH, and the cut-off score of 33.8 is useful to provide interpretable information for risk stratification. The decision regarding adequate cut-off values involves a trade-of between sensitivity and specificity. For a given test, the choice should consider its relative cost, which includes the analysis of the occurrence of false positives and false negatives [41]. Considering the mortality of high-risk patients, higher sensitivity is prioritized. In the current study, the DASI score of 33.8 has good discriminative ability for screening high-risk patients, with 91% sensitivity and 65% specificity. Based on the results, the DASI enables clinicians and patients to determine the dynamic changes of PAH easily and economically and decide whether further evaluation, such as blood biomarkers, echocardiography, and additional interventions, are required. Therefore, the DASI is a simple and valid tool for assessing the exercise capacity of patients with PAH, especially in low-resource settings.

There are several limitations in the study. Firstly, the study adopts a single-center design and was limited to the generality of the results. Secondly, the predictive ability of the DASI to risk stratification should be further proven by a longitudinal follow-up. Nevertheless, our promising results provide critical information essential for clinical utility. The patients in the study are followed up through an outpatient service and/or telephoned regularly since enrollment, which will verify our results.

## 5. Conclusions

The DASI is a valid and practical self-assessment tool for exercise capacity in patients with PAH and has a good predictive ability in risk stratification, with important clinical implications to identify patients who have poor exercise capacity and a high risk, particularly in settings with limited medical resources.

## Figures and Tables

**Table 1:** Baseline demographic and clinical characteristics of the patients.

Variable	All Patients	IPAH	CHD–PAH	Others	*p*-Value
	(n = 89)	(n = 20)	(n = 62)	(n = 7)	
Age—years	34.53 ± 8.97	36.25 ± 9.36	33.47 ± 8.58	36.29 ± 3.04	0.075
BMI—kg/m^2^	20.29 ± 3.35	22.30 ± 2.76	19.46 ± 3.30	21.37 ± 3.08	0.003
Smoking—yes/no	14 (15.7%)/75 (84.3%)	3 (15.0%)/17 (85.0%)	9 (14.5%)/53 (85.5%)	2 (28.6%)/5 (71.4)	0.623
Gender—female/male	74 (83.1%)/15 (16.9%)	16 (80.0%)/4 (20.0%)	52 (83.9%)/10 (16.1%)	6 (85.7%)/1 (14.3%)	0.909
Marital status					0.293
Married	58 (65.2%)	17 (85.0%)	35 (56.5%)	6 (85.7%)	
Single	25 (28.1%)	2 (10.0%)	22 (35.5%)	1 (14.3%)	
Divorce	6 (6.7%)	1 (5.0%)	5(8.0%)	0	
Educational level					0.373
Primary school	7 (7.8%)	3 (15.0%)	3 (4.8%)	1 (14.3%)	
Middle school	24 (27.0%)	4 (20.0%)	18 (29.0%)	2 (28.6%)	
High school	24 (27.0%)	4 (20.0%)	20 (32.3%)	0	
College or above	34 (38.2%)	9 (45.0%)	21 (33.9%)	4 (57.1%)	
Course of disease (year)	5.73 ± 4.57	4.55 ± 3.85	5.95 ± 4.82	7.29 ± 4.82	0.112
WHO-FC					0.762
I	21 (23.6%)	3 (15.0%)	16 (25.8%)	2 (28.6%)	
II	62 (69.7%)	15 (75.0%)	43 (69.4%)	4 (57.1%)	
III	6 (6.7%)	2 (10.0%)	3 (4.8%)	1 (14.3%)	
Right heart catheterization					
PAP (mean)—mmHg	57.28 ± 21.67	49.50 ± 20.03	59.70 ± 22.19	53.83 ± 19.5	0.383
3wPAWP—mmHg	8.97 ± 3.15	8.67 ± 3.11	9.02 ± 3.24	9.17 ± 3.24	0.937
CI—L/min/m^2^	3.46 ± 1.09	3.25 ± 1.08	3.46 ± 1.08	3.84 ± 1.28	0.403
RAP—mmHg	5.03 ± 3.19	7.17 ± 5.02	4.51 ± 2.47	4.67 ± 1.86	0.201
Echocardiography					
LVEF %	68.78 ± 5.74	69.10 ± 5.19	68.80 ± 5.90	67.71 ± 6.53	0.988
TAPSE mm	18.01 ± 3.84	19.84 ± 4.10	17.64 ± 3.51	16.03 ± 4.53	0.084
DASI total score	36.47 ± 13.58	33.09 ± 13.45	37.52 ± 13.39	36.84 ± 16.08	0.451
PeakVO_2_—mL/min/kg	13.70 ± 3.74	13.97 ± 2.26	13.71 ± 3.90	12.87 ± 7.74	0.890
Peak VO_2_ < 11 mL/min/kg	20 (22.4%)	4 (20.0%)	14 (22.5%)	2 (28.6%)	0.342
6MWD—meters	478.10 ± 100.80	516.45 ± 89.54	467.97 ± 103.16	467.97 ± 100.40	0.161

**Table 2:** Standardized coefficients of unary linear regression models for DASI to predict peak VO_2_ (mL/kg/min) and 6MWD (meters).

	All the PAH (n = 89)				CHD–PAH (n = 63)			
	r	r^2^	Standardization Coefficient	*p*	r	r^2^	Standardization Coefficient	*p*
DASI and PeakVO_2_	0.467	0.218	0.467	<0.001	0.515	0.265	0.515	<0.001
DASI and 6MWD	0.501	0.251	0.501	<0.001	0.605	0.366	0.605	<0.001

**Table 3:** Distribution of WHO functional classes in high-risk and non-high-risk groups according to the cut-off value of the DASI.

WHO Function Class	High-Risk (DASI < 33.8)	Non-High-Risk (DASI > 33.8)	Sig.
I	6	16	χ^2^ = 7.267 *p* = 0.001
II	32	29	
III	5	1	

## Data Availability

The data are available from the corresponding author upon reasonable request.
